# Circular RNAs in body fluids as cancer biomarkers: the new frontier of liquid biopsies

**DOI:** 10.1186/s12943-020-01298-z

**Published:** 2021-01-11

**Authors:** Sumeng Wang, Ke Zhang, Shanyue Tan, Junyi Xin, Qianyu Yuan, Huanhuan Xu, Xian Xu, Qi Liang, David C. Christiani, Meilin Wang, Lingxiang Liu, Mulong Du

**Affiliations:** 1 Department of Oncology, The First Affiliated Hospital of Nanjing Medical University, 300 Guangzhou Road, Nanjing, 210029 Jiangsu People’s Republic of China; 2 Department of Environmental Genomics, Jiangsu Key Laboratory of Cancer Biomarkers, Prevention and Treatment, Collaborative Innovation Centre for Cancer Personalized Medicine, Nanjing Medical University, Nanjing, People’s Republic of China; 3 Department of Genetic Toxicology, The Key Laboratory of Modern Toxicology of Ministry of Education, Centre for Global Health, School of Public Health, Nanjing Medical University, 101 Longmian Avenue, Nanjing, 211166 Jiangsu People’s Republic of China; 4 Departments of Environmental Health, Harvard T.H. Chan School of Public Health, 677 Huntington Avenue, Boston, MA 02115 USA; 5 Department of Medicine, Massachusetts General Hospital, 55 Fruit Street, Boston, MA 02114 USA; 6 Department of Biostatistics, Centre for Global Health, School of Public Health, Nanjing Medical University, 101 Longmian Avenue, Nanjing, 211166 Jiangsu People’s Republic of China

**Keywords:** Circular RNA, Liquid biopsy, Cancer biomarker

## Abstract

Cancer is a leading cause of death worldwide, particularly because of its high mortality rate in patients who are diagnosed at late stages. Conventional biomarkers originating from blood are widely used for cancer diagnosis, but their low sensitivity and specificity limit their widespread application in cancer screening among the general population. Currently, emerging studies are exploiting novel, highly-accurate biomarkers in human body fluids that are obtainable through minimally invasive techniques, which is defined as liquid biopsy. Circular RNAs (circRNAs) are a newly discovered class of noncoding RNAs generated mainly by pre-mRNA splicing. Following the rapid development of high-throughput transcriptome analysis techniques, numerous circRNAs have been recognized to exist stably and at high levels in body fluids, including plasma, serum, exosomes, and urine. CircRNA expression patterns exhibit distinctly differences between patients with cancer and healthy controls, suggesting that circRNAs in body fluids potentially represent novel biomarkers for monitoring cancer development and progression. In this study, we summarized the expression of circRNAs in body fluids in a pan-cancer dataset and characterized their clinical applications in liquid biopsy for cancer diagnosis and prognosis. In addition, a user-friendly web interface was developed to visualize each circRNA in fluids (https://mulongdu.shinyapps.io/circrnas_in_fluids/).

**Supplementary Information:**

The online version contains supplementary material available at 10.1186/s12943-020-01298-z.

## Background

Cancer is an important public health issue worldwide and the second leading cause of death in the United States [1]. Cancer arises from genetic alterations and dysregulated transcriptional programmes [2]. In recent years, cancer has imposed a tremendous burden on individuals, families, communities and health systems [3]. However, the early detection of cancer can help to minimize the cancer burden [4]. Over the past few decades, several blood-based biomarkers [e.g., carcinoembryonic antigen (CEA) and prostate-specific antigen (PSA)] have been used for the early detection of cancers, but the lack of sensitive and specific biomarkers has limited the early diagnosis and determination of the prognosis of many patients with cancer [4]. For example, PSA is present in both patients with prostate cancer and patients with benign prostate hyperplasia [5]. Thus, studies of biomarkers with high specificity and sensitivity are urgently needed.

Liquid biopsy is a non-invasive method that uses body fluids, such as blood, plasma, serum, urine, and gastric juice, to reflect the disease state [6]. Recently, substantial attention has been devoted to detecting and quantifying biomarkers, especially circular RNAs (circRNAs), in tumour biopsies [7]. Although the study on circRNAs is in its infancy, several studies have revealed their potential as valuable diagnostic and prognostic biomarkers for cancers [8]. CircRNAs are highly resistant to RNase activity because of the lack of 5′ and 3′ ends [9–11]. In addition, circRNAs are ideal candidates as liquid biopsy biomarkers, as they are often expressed in tissue- and developmental stage-specific manners, and are found in large quantities not only in tissues and cells but also in body fluids [8, 12].

In this review, we summarized the expression pattern of circRNAs in body fluids across pan-cancers and characterized their clinical applications in liquid biopsy. The receiver operating characteristic (ROC) curves and the corresponding area under the curve (AUC) values extracted from each analysis were used to describe the diagnostic value of the circRNAs. In addition, we developed a user-friendly web interface that enabled interested individuals to easily browse and search for disease-related circRNAs.

## Cancer biomarkers for liquid biopsies

Liquid biopsy, a measurement of monitoring tumours in real-time, is a non-invasive biopsy method that has been investigated by scientists and oncologists for several years [13, 14]. The aim of liquid biopsy is to identify and quantify the tumour-derived biological materials circulating in body fluids [15]. Several researchers have described the advantages of liquid biopsy compared with solid biopsy [16–18]. First, liquid biopsy detects the presence of a tumour at an earlier stage before it has been detected by radiological and imaging examinations [16, 19]. Second, liquid biopsies may better characterize the whole tumour, not just a small area, which is a particularly important consideration for histologically heterogeneous tumours; in contrast, classical solid biopsies, which analyse only specific sections of malignancies, might ignore important molecular traits [16, 20]. Third, the possibility of repeated sampling via liquid biopsy allows more accurate and more dynamic monitoring of disease progression and modulation of the treatment strategy [16, 21]. Moreover, liquid biopsy is a convenient way to monitor progression and recurrence early during patient follow-up [21, 22]. In conclusion, liquid biopsy may support the “gold standard” tissue biopsy or even replace tissue biopsy in the future.

## Biological characteristics of circRNAs

**Fig. 1:**
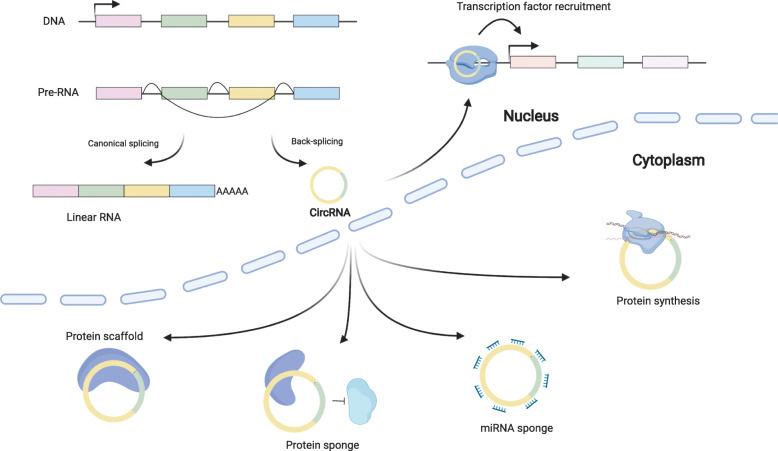
The potential biogenetic and functional mechanisms of circRNAs. Schematic showing the biogenesis of circRNAs through the noncanonical back-splicing process and their reported functional mechanisms. CircRNAs are generated by a process called back-splicing from linear pre-messenger RNA. During this process, the 3′ and 5′ ends are ligated to form a covalently closed, uninterrupted loop. Unlike linear RNAs, circRNAs are relatively stable and not easily degraded by endonucleases. CircRNAs function as miRNA sponges, bind RBPs, and are translated due to the existence of an internal ribosome entry site. This schematic was generated at https://app.biorender.com/gallery/illustrations

When circRNAs were first discovered in the early 1970s, they were thought to be by-products of splicing without any valuable biological functions [23]. However, with the emergence of next-generation sequencing technology and the development of bioinformatics pipelines, numerous circRNAs have recently been discovered to be differentially expressed among patients with various cancers [7, 24]. During the process named back-splicing, circRNAs are generated from linear pre-messenger RNAs, and the 3′ and 5′ ends are ligated to form a covalently closed, uninterrupted loop [24]. These closed-loop structures originate from the ligation of introns, exons, or both [7, 25]. The potential biogenetic and functional mechanisms of circRNAs are presented in Fig. 1.

Several studies have shown that most circRNAs contain miRNA response elements (MREs), functioning as miRNA sponges [26]. Moreover, circRNAs have been reported to increase the expression of innumerable mRNAs through an indirect mechanism [27]. CircRNAs can also bind RNA binding proteins (RBPs), such as MBL [28], SR proteins [29], and IGF2BP3 [7, 30]. In addition, circRNAs can be translated due to the presence of an internal ribosome entry site (IRES) in the sequence [31].

## CircRNAs in body fluids from a pan-cancer dataset as cancer biomarkers

**Fig. 2:**
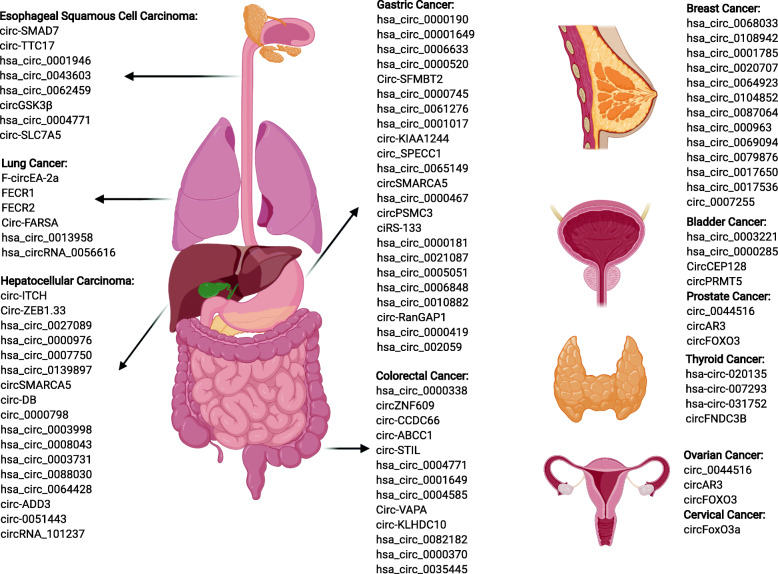
CircRNAs identified in patients with the 10 most common cancers. The ten most common cancers are shown according to “cancer statistics in 2020”[1], including lung cancer, breast cancer, colorectal cancer, prostate cancer, gastric cancer, hepatocellular carcinoma, esophageal squamous cell carcinoma, cervical cancer, thyroid cancer, and bladder cancer. Ovarian cancer-related circRNAs are listed in this figure because of its increasing morbidity and mortality rates in females. This figure was generated at https://app.biorender.com/gallery/illustrations

**Fig. 3:**
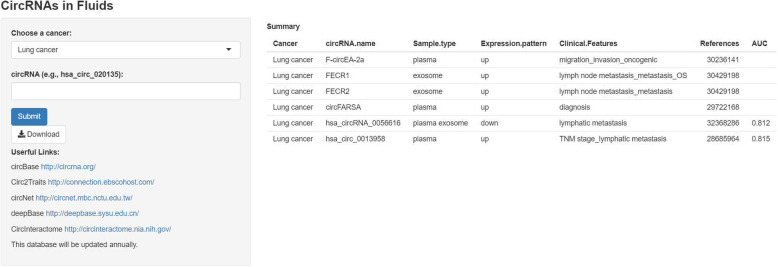
A user-friendly interface to visualize the database of circRNAs in body fluids. The *shiny* package in R was utilized to develop this interface, and this interface is accessible in a user-friendly format at https://mulongdu.shinyapps.io/circrnas_in_fluids/. Moreover, some useful links are listed in this interface and users can click them to obtain more information. More detailed information can be acquired by navigating to this website or clicking on the hyperlink in the electronic version of this manuscript

Recently, many circRNAs that function as oncogenes or tumour-suppressors have been discovered to be dysregulated in tumour specimens. Based on emerging evidence, circRNAs are associated with cancer progression and are involved in the malignant cellular behaviours [32–35]. As the shape of circRNAs is a covalently closed continuous loop, they have relatively stable framework in the eukaryotic transcriptome [36, 37]. Unlike linear RNAs, circRNAs are relatively stable and are not easily degraded by endonucleases [36, 38–40]. Here, we summarize recent studies on circRNAs in body fluids, including 112 differentially expressed circRNAs identified in various cancers from a search of PubMed for studies published up to 15 May, 2020 with the following key words: “circular RNA” OR “circular RNAs” OR “circRNA” OR “circRNAs” AND “cancer” AND (“liquid biopsies” OR “biomarker” OR “non-invasive”). Detailed information about these 112 circRNAs is presented in Supplementary Figure 1 and Supplementary Table 1. Figure 2 shows the circulating circRNAs identified in the ten most common cancers. In addition, a new intuitive web interface for users to explore and analyse circRNAs was developed using the *shiny* package in R (Fig. 3), which is easily accessed at https://mulongdu.shinyapps.io/circrnas_in_fluids/.

### Lung cancer

Lung cancer is the leading cause of cancer-related morbidity and mortality worldwide, of which non-small cell lung cancer (NSCLC) accounts for more than 80% of lung cancer cases [1, 41]. CircFARSA, derived from exons 5–7 of *FARSA* gene, was present at higher levels in plasma from patients than from healthy controls, indicating that plasma circFARSA can act as a potential non-invasive diagnostic biomarker for lung cancer [42]. Tan et al. observed that the suppression of cell migration and invasion after silencing F-circEA-2a [43]. Zhu et al. identified plasma hsa_circ_0013958 as a functional diagnostic and prognostic biomarker, because its presence in plasma from patients with lung cancer positively correlated with the TNM stage and lymphatic metastasis, with an AUC of 0.815, and it could promote cell proliferation and invasion as well as inhibit apoptosis [32]. Additionally, serum exosomal circRNAs FECR1 and FECR2 played important roles in lung cancer progression [44].

### Breast cancer

Breast cancer is the second most frequently diagnosed cancer and the most common cancer in females [1, 41]. The plasma level of hsa_circ_0001785 was closely correlated with the histological grade, TNM stage, and distant metastasis of breast cancer, with higher diagnostic value (AUC = 0.784) than both CEA (AUC = 0.562) and carbohydrate antigen 153 (CA153) (AUC = 0.629) [45]. In addition, hsa_circ_0068033 and hsa_circ_0108942 in plasma had potential diagnostic value in the clinic [45]. Wang et al. reported the significant overexpression of hsa_circ_0020707, hsa_circ_0064923, hsa_circ_0104852, hsa_circ_0087064, and hsa_circ_0009634 in the serum from patients with breast cancer and positive correlations with carcinogenesis and progression [46]. Additionally, Jia et al. documented higher levels of circ_0007255 in serum from patients compared to serum from healthy controls [47].

### Colorectal cancer

Colorectal cancer is the third most common cancer worldwide [1, 41]. Three circRNAs (circ-CCDC66, circ-ABCC1, and circ-STIL) were significantly downregulated in plasma from patients with colorectal cancer compared to healthy controls, and all of these circRNAs presented higher AUC values than those of commonly used cancer biomarkers, such as CEA and carbohydrate antigen 19–9 (CA19–9) [48]. Exosomal hsa_circ_0004771 and circ-KLHDC10 were significantly upregulated in patients with colorectal cancer, suggesting that they may represent potentially novel diagnostic biomarkers for colorectal cancer [49, 50]. Tian et al. discovered that hsa_circ_0004585 was positively related to tumour size and involved in colorectal cancer carcinogenesis and metastasis; moreover, hsa_circ_0004585 derived from peripheral samples had an AUC of 0.707, with a sensitivity and specificity of 0.908 and 0.408, respectively, indicating its clinical importance in diagnosing colorectal cancer [34]. A set of three circRNAs (hsa_circ_0000370, hsa_circ_0082812, and hsa_circ_0035445) were found to be dysregulated in colorectal cancer plasma, with AUCs of 0.815, 0.737, and 0.703, respectively [51]. Li et al. verified that the increased plasma circVAPA (hsa_circ_0006990) levels could exert oncogenic effects by sponging miR-101 to affect colorectal cancer development [52]. The expression of circZNF609 was downregulated in the serum from patients with colorectal cancer [53]; and hsa_circ_0001649 showed a close relation to pathological differentiation, with an AUC of 0.857 [54].

### Prostate cancer

Prostate cancer is the fourth most frequently diagnosed cancer, with a high cancer-related mortality rate worldwide [1, 41]. Exosomal circ_0044516 was significantly upregulated in patients with prostate cancer and regulated the proliferation and metastasis of cancer cells by modulating miR-29a-3p expression [55]. Kong et al. reported the upregulation of circFOXO3 (hsa_circ_0006404), which was derived from exon 2 of the forkhead box O3 (*FOXO3*) gene, in serum from patients with prostate cancer; circFOXO3 exhibited oncogenic activity by altering the cell cycle and apoptosis in the process of sponging miR-29a-3p to regulate *SLC25A15* expression [33].

### Gastric cancer

Gastric cancer is the fifth most frequently diagnosed malignancy and the third leading cause of cancer-related death worldwide [1, 41]. Twenty-three circRNAs have been discovered to be dysregulated in patients with gastric cancer, and 18 of which were downregulated and 5 were upregulated; detailed information is presented in Supplementary Table 1. Plasma hsa_circ_0000419 level was significantly associated with advanced tumour stage, lymphatic and distal metastasis, as well as venous and perineural invasion of gastric cancer, serving as a prognostic indicator and tumour suppressor [35]. The plasma level of hsa_circ_0000190 was decreased in patients with gastric cancer, with a high AUC of 0.600, and was associated with the tumour diameter, TNM stage, lymphatic metastasis, distal metastasis, and CA19–9 level, indicating that hsa_circ_0000190 could serve as a novel non-invasive diagnostic biomarker [56]. The low plasma exosomal hsa_circ_0065149 level in patients with early stage gastric cancer suggested that hsa_circ_0065149 would be a useful indicator for the early screening of gastric cancer [57]. Rong et al. observed significant correlations between a lower expression level of circPSMC3 in patients with gastric cancer and a higher TNM stage and shorter overall survival (OS) time; and circPSMC3 could participate in the progression of gastric cancer by sponging miRNA-296-5p to regulate the expression of Phosphatase and Tensin Homolog (*PTEN*), providing new insights into the treatment of gastric cancer [58]. Hsa_circ_0000467 was noticeably overexpressed in plasma from patients with gastric cancer, with an AUC of 0.790, a value that was higher than those of traditional cancer biomarkers, such as CEA and carbohydrate antigen 724 (CA-724). In terms of biological function, knockdown of hsa_circ_0000467 markedly inhibited the proliferation, invasion, and migration of gastric cancer cells, indicating that hsa_circ_0000467 could play a functional role as a non-invasive diagnostic and prognostic biomarker for gastric cancer [59].

### Hepatocellular carcinoma

Hepatocellular carcinoma (HCC), the sixth most common cause of cancer-related death worldwide, accounts for approximately 80% of primary liver cancers [1, 41]. Chen et al. discovered a significantly lower expression level of circ_0051443 in plasma exosomes from patients than that from healthy controls, serving as a predictive and diagnostic biomarker, and it could suppress malignant biological behaviours by regulating apoptosis, proliferation, and cell cycle arrest [60]. In addition, the plasma level of hsa_circ_0027089, hsa_circ_0000976, hsa_circ_0007750, and hsa_circ_0139897 have been used to distinguish patients with HCC from healthy controls [61, 62]. The plasma level of hsa_circ_0003998 in healthy controls was significantly lower than that in patients with HCC, with an AUC value of 0.892 alone and 0.947 in combination with alpha fetoprotein (AFP). In addition, patients with lower plasma hsa_circ_0003998 levels experienced a prolonged OS compared with patients with higher levels, indicating that hsa_circ_0003998 represented a potential diagnostic and prognostic biomarker for HCC [63]. A lower expression level of circSMARCA5 has been observed in plasma from patients with HCC than that from healthy controls with high discriminatory accuracy (AUC = 0.938), serving as a biomarker for distinguishing patients with HCC from healthy controls [64]. Weng et al. reported correlations between plasma hsa_circ_0064428 and HCC survival, tumour size, and metastasis, suggesting a role as a prognostic biomarker for HCC [65]. Serum circ-ZEB1.33 was associated with the TNM stage and prognosis, and this circRNA functioned as a miR-200a-3p sponge to upregulate *CDK6* [66]. Moreover, low-expressed circ-ITCH [67] and circ-ADD3 [68], as well as high-expressed circRNAs (hsa_circ_0008043 [69], hsa_circ_0003731 [69], hsa_circ_0088030 [69], circ-DB [70], and circ_0000798 [71]), played potential prognostic roles in patients with HCC.

### Esophageal squamous cell carcinoma

Esophageal squamous cell carcinoma (ESCC) has high incidence and mortality rates, and ranks the seventh among the most frequently diagnosed malignancies worldwide [1, 41]. Plasma levels of hsa_circ_00021946 and hsa_circ_0043603 detected were used as diagnostic biomarkers [72]. Plasma levels of circ-SMAD7 were significantly lower in patients with ESCC than that in normal controls and correlated with the TNM stage and lymphatic metastasis, serving as a novel diagnostic biomarker for ESCC [73]. Wang et al. demonstrated that upregulation of circ-TTC17 in patient plasma was correlated with the proliferation and migration of ESCC cells, proving that circ-TTC17 had potential prognostic value for ESCC [74]. In addition, the expression level of circGSK3β could be as a novel non-invasive biomarker for detecting ESCC and early stage ESCC with AUCs of 0.782 and 0.793, respectively [75]. Upregulation of hsa_circ_0004771 in plasma was associated with a heavier tumour burden and poor prognosis, and hsa_circ_0004771 knock down could inhibit ESCC cell proliferation via the miR-339-5p/*CDC25A* axis [76]. Wang et al. discovered a correlation between high plasma levels of circ-SLC7A5 in patients with a high TNM stage, suggesting a role as a prognostic biomarker [77].

### Cervical cancer

Cervical cancer ranks eighth among the most frequently diagnosed cancers worldwide, and it is the fourth most frequent and important cause of cancer-related mortality in women [1, 41]. CircFoxO3a, which was downregulated in patient serum, was associated with deeper stromal invasion and positive lymph node metastasis; in addition, patients with higher expression levels of serum circFoxO3a would have better prognoses [78].

### Thyroid cancer

Thyroid cancer is the ninth most common cancer worldwide [1, 41]. Wu et al. documented the high expression of circFNDC3B in serum exosomes from patients with thyroid cancer, and this circRNA modulated thyroid cancer progression via the miR-1178/*TLR4* pathway [79].

### Bladder cancer

Bladder cancer ranks tenth among the commonly diagnosed cancers, with a high recurrence rate worldwide [1, 41]. Blood hsa_circ_0003221 was significantly associated with clinicopathological characteristics, including poor differentiation, lymph node metastasis, and high T stage; and was involved in the inhibition of cell proliferation and migration [80]. Chi et al. discovered the low expression level of hsa_circ_0000285 in serum from patients with bladder cancer was associated with tumour size, tumour differentiation, distant metastasis, and TNM stage, indicating that hsa_circ_0000285 represented a novel prognostic biomarker for bladder cancer [81]. In addition, circPRMT5 was upregulated in serum and urinary exosomes from patients with bladder cancer and exhibited a strong correlation with tumour metastasis [82].

### Ovarian cancer

Recently, the morbidity and mortality rates of ovarian cancer have increased annually [1, 41]. Hu et al. observed the downregulation of circBNC2 in plasma samples from patients with ovarian cancer compared with that from healthy controls [83]. The high serum circSETDB1 levels identified in patients with ovarian cancer were significantly increased in patients with primary chemoresistance, which could distinguish patients with ovarian cancer from healthy controls and patients with primary chemoresistance from those with primary chemosensitivity; these findings identified a role for serum circSETDB1 levels as a diagnostic and prognostic biomarker [84]. In addition, Cdr1as, which was involved in the mechanism regulating the miR-1270/*SCAI* signalling pathway, was expressed at low levels in serum exosomes from patients with cisplatin resistance [85].

### Other common tumours

In addition to these top cancers, circRNAs are also involved in the tumorigenesis of many other cancers. For example, hsa_circ_0081001 was overexpressed in serum from patients with osteosarcoma and exhibited a better diagnostic and prognostic value than either alkaline phosphatase (ALP) or lactate dehydrogenase (LDH) [86]; increased serum hsa_circ_0000885 levels showed a similar diagnostic value [87]. Higher plasma levels of circ-LDLRAD3 have been detected in patients with pancreatic cancer than those in healthy controls, and were associated with venous invasion, lymphatic invasion, and metastasis, with an AUC of 0.670 alone and a higher AUC of 0.870 in combination with CA19–9 [88]. In patients with nasopharyngeal carcinoma, serum levels of circRNA_0000285 were increased and significantly correlated with the differentiation grade, tumour size, lymph node metastasis, distal metastasis, and TNM stage, indicating an independent prognostic indicator for nasopharyngeal carcinoma [89]. In addition, hsa_circ_0109046 and hsa_circ_0002577 were upregulated in the serum of patients with endometrial cancer [90], and exosomal circ-0000284 was overexpressed in patients with cholangiocarcinoma [91].

## Conclusions and future perspectives

In this review, we comprehensively summarized clinically valuable circRNAs in body fluids as diagnostic and prognostic cancer biomarkers.

Due to its high morbidity and mortality rates, cancer is a major threat to human health [2]. Accounting for the state-of-the-art detection technologies, including next-generation sequencing, an increasing number of circRNAs that are differentially expressed in different clinical stages have been examined and quantified [7, 24]. Compared to conventional cancer biomarkers (e.g. PSA and CEA), circRNAs have higher sensitivity and specificity in diagnosis and prognosis, and play an increasingly important role in the oncogenesis and progression of diverse tumours [48, 59].

CircRNAs have various biological mechanisms, including the mainly reported function of miRNA sponges, which could increase the complexity of the competing endogenous RNA (ceRNA) network. In addition, studies exploring the other biological functions of circRNAs in tumours, such as their roles as gene regulators, should not be ignored [92–95].

Currently, many studies have identified that circRNAs as potentially novel diagnostic and prognostic biomarkers for multiple tumours. Although the detection of circRNAs has mainly been conducted in tissues and cell lines, more studies have focused on circRNAs detection using less invasive and more accessible methods, such as liquid biopsy [6, 15, 16]. Moreover, combinations of circRNAs with traditional cancer biomarkers may exhibit higher diagnostic or prognostic accuracy than single traditional biomarkers [63].

Different from previous reviews about circRNA-miRNA-mRNA axis that have mainly described biological functions in tumour tissues or cells, we reviewed the expression of circRNAs in body fluids from patients with various tumours and summarized their diagnostic and prognostic values as non-invasive biomarkers in clinical applications; furthermore, we made a user-friendly interface for readers to obtain the corresponding data [8, 96–98].

We have to acknowledge that difficulties and challenges of circRNAs existed in clinical applications [22]. First, circRNAs are difficult to accurately detect and recognize in body fluids due to their low abundance [99]. Second, the detection methods must to be improved to enable increased precision in detecting circRNAs [99]. Thus, we should improve advanced technologies and experimental approaches to apply circRNAs in clinical practice.

Research into circRNAs as cancer biomarkers is still in its infancy, and little is known about their mechanisms in tumorigenesis and tumour progression. Because of the substantial improvements that are ongoing, we propose that most of these limitations will be overcome and that more circRNAs will be detected and applied in the clinic in the future.

## Supplementary Information

**Additional file 1: Figure S1**. The total number of circRNAs in the pan-cancer dataset. **Supplementary Table 1**. Summary of the expression pattern and sample type of circRNAs involved in pan-cancer

## Data Availability

All data were available and accessible in web (https://mulongdu.shinyapps.io/circrnas_in_fluids/).

## Abbreviations

circRNAs: circular RNAs
PSA: Prostate specific antigen
ROC curve: Receiver operating characteristic curve
AUC: The area under ROC curve
MREs: miRNA response elements
RBPs: RNA binding proteins
IRES: Internal ribosome entry site
NSCLC: Non-small cell lung cancer
CEA: Carcinoembryonic antigen
CA19–9: Carbohydrate antigen 19–9
CA153: Carbohydrate antigen 153
*FOXO3*: Forkhead box O3
*PTEN*: Phosphatase and Tensin Homolog
CA-724: Carbohydrate antigen 724
HCC: Hepatocellular carcinoma
AFP: Alpha fetoprotein
ESCC: Esophageal squamous cell carcinoma
ALP: Alkaline phosphatase
LDH: Lactate dehydrogenase
ceRNA: competing endogenous RNA
OS: Overall survival
