# Supplementary material for: Circular RNAs in body fluids as cancer biomarkers: the new frontier of liquid biopsies
Source: Mol Cancer. 2021 Jan 11;20:13. doi: 10.1186/s12943-020-01298-z (PMC7798340; doi:10.1186/s12943-020-01298-z)
Supplement: Supplementary file 1 — Additional file 1: Figure S1. The total number of circRNAs in the pan-cancer dataset. Supplementary Table 1. Summary of the expression pattern and sample type of circRNAs involved in pan-cancer [file 12943_2020_1298_MOESM1_ESM.docx]

**Supplementary Figure Legends**

**Figure S1. The total number of circRNAs in the pan-cancer dataset.**

A total of 112 circRNAs are presented in descending order in this bar chart. The highest number of these circRNAs were found in gastric cancer.

**Fig. S1**


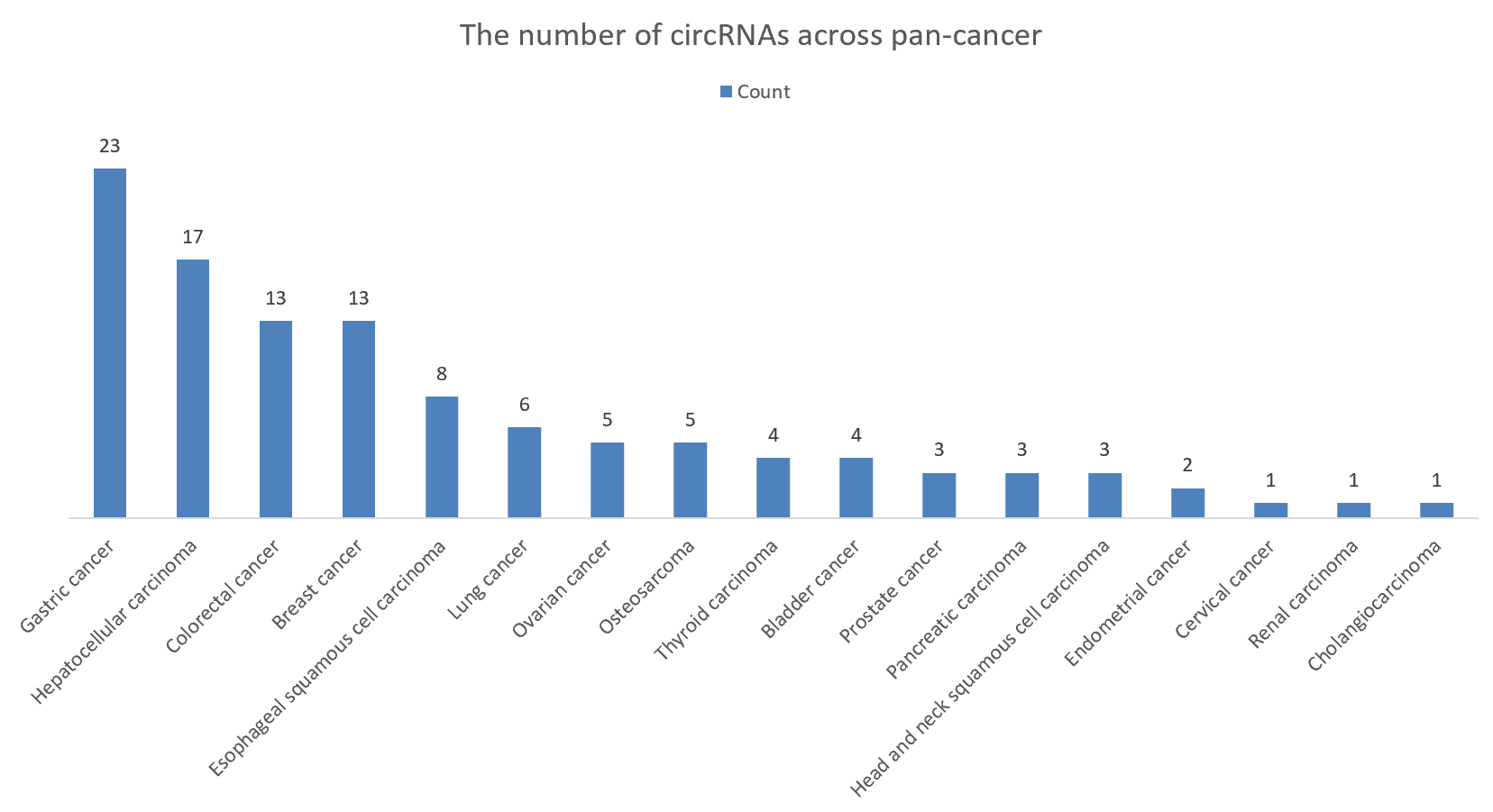


**Supplementary Table 1. Summary of the expression pattern and sample type of circRNAs involved in pan-cancer.**

| **Cancer** | **CircRNAs Name** | **Sample Type** | **Expression pattern** | **References** |
| --- | --- | --- | --- | --- |
| **Lung Cancer** | F-circEA-2a | plasma | up | (1) |
|  | FECR1 | exosome | up | (2) |
|  | FECR2 | exosome | up | (2) |
|  | circFARSA | plasma | up | (3) |
|  | hsa_circ_0013958 | plasma | up | (4) |
|  | hsa_circRNA_0056616 | plasma exosome | down | (5) |
| **Breast Cancer** | hsa_circ_0068033 | plasma | down | (6) |
|  | hsa_circ_0108942 | plasma | up | (6) |
|  | hsa_circ_0001785 | plasma | up | (6) |
|  | hsa_circ_0020707 | serum | up | (7) |
|  | hsa_circ_0064923 | serum | up | (7) |
|  | hsa_circ_0104852 | serum | up | (7) |
|  | hsa_circ_0087064 | serum | up | (7) |
|  | hsa_circ_0009634 | serum | up | (7) |
|  | hsa_circ_0069094 | plasma | up | (8) |
|  | hsa_circ_0079876 | plasma | up | (8) |
|  | hsa_circ_0017650 | plasma | up | (8) |
|  | hsa_circ_0017536 | plasma | up | (8) |
|  | circ_0007255 | serum | up | (9) |
| **Colorectal Cancer** | hsa_circ_0000338 | serum exosome | down | (10) |
|  | circZNF609 | serum | down | (11) |
|  | circ-CCDC66 | plasma | down | (12) |
|  | circ-ABCC1 | plasma | down | (12) |
|  | circ-STIL | plasma | down | (12) |
|  | hsa-circ-0004771 | serum | up | (13) |
|  | hsa_circ_0001649 | serum | down | (14) |
|  | circVAPA | plasma | up | (15) |
|  | circ-KLHDC10 | serum exosome | up | (16) |
|  | hsa_circ_0082182 | plasma | up | (17) |
|  | hsa_circ_0000370 | plasma | up | (17) |
|  | hsa_circ_0004585 | plasma | up | (18) |
|  | hsa_circ_0035445 | plasma | down | (17) |
| **Prostate Cancer** | circ_0044516 | exosome | up | (19) |
|  | circAR3 | plasma | up | (20) |
|  | circFOXO3 | plasma | up | (21) |
| **Gastric Cancer** | hsa_circ_0000190 | plasma | down | (22) |
|  | hsa_circ_0065149 | plasma, gastric juice | down | (23) |
|  | hsa_circ_00001649 | serum | down | (24) |
|  | hsa_circ_0006633 | plasma | down | (25) |
|  | hsa_circ_0000520 | plasma | down | (26) |
|  | circ-KIAA1244 | plasma | down | (27) |
|  | circ-SFMBT2 | plasma | up | (28) |
|  | hsa_circ_0000745 | plasma | down | (29) |
|  | hsa_circ_0061276 | plasma | down | (30) |
|  | hsa_circ_0001017 | plasma | down | (30) |
|  | circ_SPECC1 | plasma | down | (31) |
|  | circSMARCA5 | plasma | down | (32) |
|  | hsa_circ_0000467 | plasma | up | (33) |
|  | circPSMC3 | plasma | down | (34) |
|  | ciRS-133 | plasma, exosome | up | (35) |
|  | hsa_circ_0000181 | plasma | down | (36) |
|  | hsa_circ_0021087 | plasma | down | (37) |
|  | hsa_circ_0005051 | plasma | down | (37) |
|  | hsa_circ_0006848 | plasma | down | (38) |
|  | hsa_circ_0010882 | plasma | up | (39) |
|  | circ-RanGAP1 | plasma exosomes | up | (40) |
|  | hsa_circ_0000419 | plasma | down | (41) |
|  | hsa_circ_002059 | plasma | down | (42) |
| **Hepatocellular Carcinoma** | circ-ITCH | plasma | down | (43) |
|  | circ-ZEB1.33 | serum | up | (44) |
|  | hsa_circ_0027089 | plasma | up | (45) |
|  | hsa_circ_0000976 | plasma | up | (46) |
|  | hsa_circ_0007750 | plasma | up | (46) |
|  | hsa_circ_0139897 | plasma | up | (46) |
|  | circSMARCA5 | plasma | down | (47) |
|  | circ-DB | plasma exosomes | up | (48) |
|  | circ_0000798 | PBMCs | up | (49) |
|  | hsa_circ_0003998 | plasma | up | (50) |
|  | hsa_circ_0008043 | serum exosomes | up | (51) |
|  | hsa_circ_0003731 | serum exosomes | up | (51) |
|  | hsa_circ_0088030 | serum exosomes | up | (51) |
|  | circ-ADD3 | plasma | down | (52) |
|  | circ-0051443 | plasma exosome | down | (53) |
|  | hsa_circ_0064428 | plasma | down | (54) |
|  | circRNA_101237 | serum | up | (55) |
| **Esophageal Squamous Cell Carcinoma** | circ-SMAD7 | plasma | down | (56) |
|  | circ-TTC17 | plasma | up | (57) |
|  | hsa_circ_0001946 | plasma | down | (58) |
|  | hsa_circ_0043603 | plasma | down | (58) |
|  | hsa_circ_0062459 | plasma | up | (58) |
|  | circGSK3β | plasma | up | (59) |
|  | hsa_circ_0004771 | plasma | up | (60) |
|  | circ-SLC7A5 | plasma | up | (61) |
| **Cervical Cancer** | circFoxO3a | serum | down | (62) |
| **Thyroid Cancer** | hsa-circ-020135 | serum exosomes | down | (63) |
|  | hsa-circ-007293 | serum exosomes | up | (63) |
|  | hsa-circ-031752 | serum exosomes | up | (63) |
|  | circFNDC3B | serum exosomes | up | (64) |
| **Bladder Cancer** | hsa_circ_0003221 | blood | up | (65) |
|  | hsa_circ_0000285 | serum | down | (66) |
|  | CircCEP128 | blood | up | (67) |
|  | circPRMT5 | serum, urine exosomes | up | (68) |
| **Ovarian Cancer** | hsa_circ_0002711 | serum | up | (69) |
|  | hsa_circ_0001756 | serum | up | (69) |
|  | circBNC2 | blood | down | (70) |
|  | circSETDB1 | serum | up | (71) |
|  | Cdr1as | plasma exosomes | down | (72) |
| **Osteosarcoma** | hsa_circ_0000190 | plasma | down | (73) |
|  | circ_HIPK3 | plasma | down | (74) |
|  | hsa_circ_0081001 | serum | up | (75) |
|  | circPVT1 | serum | up | (76) |
|  | hsa_circ_0000885 | serum | up | (77) |
| **Pancreatic Carcinoma** | circ-LDLRAD3 | plasma | up | (78) |
|  | circ-IARS | plasma | up | (79) |
|  | circ-PDE8A | plasma | up | (80) |
| **Head and Neck Squamous Cell Carcinoma** | circRNA_0000285 | serum | up | (81) |
|  | circMORC3 | plasma | down | (82) |
|  | hsa_circRNA_001387 | blood | up | (83) |
| **Endometrial Cancer** | hsa_circ_0109046 | serum | up | (84) |
|  | hsa_circ_0002577 | serum | up | (84) |
| **Cholangiocarcinoma** | circ-0000284 | exosome | up | (85) |
| **Renal Carcinoma** | hsa_circ_0039569 | blood | up | (86) |

**Supplementary references**

1. Tan S, Sun D, Pu W, Gou Q, Guo C, Gong Y, et al. Circular RNA F-circEA-2a derived from EML4-ALK fusion gene promotes cell migration and invasion in non-small cell lung cancer. Mol Cancer. 2018;17(1):138.

2. Li L, Li W, Chen N, Zhao H, Xu G, Zhao Y, et al. FLI1 Exonic Circular RNAs as a Novel Oncogenic Driver to Promote Tumor Metastasis in Small Cell Lung Cancer. Clin Cancer Res. 2019;25(4):1302-17.

3. Hang D, Zhou J, Qin N, Zhou W, Ma H, Jin G, et al. A novel plasma circular RNA circFARSA is a potential biomarker for non-small cell lung cancer. Cancer Med. 2018;7(6):2783-91.

4. Zhu X, Wang X, Wei S, Chen Y, Chen Y, Fan X, et al. hsa_circ_0013958: a circular RNA and potential novel biomarker for lung adenocarcinoma. FEBS J. 2017;284(14):2170-82.

5. He F, Zhong X, Lin Z, Lin J, Qiu M, Li X, et al. Plasma exo-hsa_circRNA_0056616: A potential biomarker for lymph node metastasis in lung adenocarcinoma. J Cancer. 2020;11(14):4037-46.

6. Yin WB, Yan MG, Fang X, Guo JJ, Xiong W, Zhang RP. Circulating circular RNA hsa_circ_0001785 acts as a diagnostic biomarker for breast cancer detection. Clin Chim Acta. 2018;487:363-8.

7. Wang J, Zhang Q, Zhou S, Xu H, Wang D, Feng J, et al. Circular RNA expression in exosomes derived from breast cancer cells and patients. Epigenomics. 2019;11(4):411-21.

8. Li Z, Chen Z, Hu G, Zhang Y, Feng Y, Jiang Y, et al. Profiling and integrated analysis of differentially expressed circRNAs as novel biomarkers for breast cancer. J Cell Physiol. 2020.

9. Jia Q, Ye L, Xu S, Xiao H, Xu S, Shi Z, et al. Circular RNA 0007255 regulates the progression of breast cancer through miR-335-5p/SIX2 axis. Thorac Cancer. 2020;11(3):619-30.

10. Hon KW, Ab-Mutalib NS, Abdullah NMA, Jamal R, Abu N. Extracellular Vesicle-derived circular RNAs confers chemoresistance in Colorectal cancer. Sci Rep. 2019;9(1):16497.

11. Zhang X, Zhao Y, Kong P, Han M, Li B. Expression of circZNF609 is Down-Regulated in Colorectal Cancer Tissue and Promotes Apoptosis in Colorectal Cancer Cells by Upregulating p53. Med Sci Monit. 2019;25:5977-85.

12. Lin J, Cai D, Li W, Yu T, Mao H, Jiang S, et al. Plasma circular RNA panel acts as a novel diagnostic biomarker for colorectal cancer. Clin Biochem. 2019;74:60-8.

13. Pan B, Qin J, Liu X, He B, Wang X, Pan Y, et al. Identification of Serum Exosomal hsa-circ-0004771 as a Novel Diagnostic Biomarker of Colorectal Cancer. Front Genet. 2019;10:1096.

14. Ji W, Qiu C, Wang M, Mao N, Wu S, Dai Y. Hsa_circ_0001649: A circular RNA and potential novel biomarker for colorectal cancer. Biochem Biophys Res Commun. 2018;497(1):122-6.

15. Li XN, Wang ZJ, Ye CX, Zhao BC, Huang XX, Yang L. Circular RNA circVAPA is up-regulated and exerts oncogenic properties by sponging miR-101 in colorectal cancer. Biomed Pharmacother. 2019;112:108611.

16. Li Y, Zheng Q, Bao C, Li S, Guo W, Zhao J, et al. Circular RNA is enriched and stable in exosomes: a promising biomarker for cancer diagnosis. Cell Res. 2015;25(8):981-4.

17. Ye DX, Wang SS, Huang Y, Chi P. A 3-circular RNA signature as a noninvasive biomarker for diagnosis of colorectal cancer. Cancer Cell Int. 2019;19:276.

18. Tian J, Xi X, Wang J, Yu J, Huang Q, Ma R, et al. CircRNA hsa_circ_0004585 as a potential biomarker for colorectal cancer. Cancer Manag Res. 2019;11:5413-23.

19. Li T, Sun X, Chen L. Exosome circ_0044516 promotes prostate cancer cell proliferation and metastasis as a potential biomarker. J Cell Biochem. 2020;121(3):2118-26.

20. Luo J, Li Y, Zheng W, Xie N, Shi Y, Long Z, et al. Characterization of a Prostate- and Prostate Cancer-Specific Circular RNA Encoded by the Androgen Receptor Gene. Mol Ther Nucleic Acids. 2019;18:916-26.

21. Kong Z, Wan X, Lu Y, Zhang Y, Huang Y, Xu Y, et al. Circular RNA circFOXO3 promotes prostate cancer progression through sponging miR-29a-3p. J Cell Mol Med. 2020;24(1):799-813.

22. Chen S, Li T, Zhao Q, Xiao B, Guo J. Using circular RNA hsa_circ_0000190 as a new biomarker in the diagnosis of gastric cancer. Clin Chim Acta. 2017;466:167-71.

23. Shao Y, Tao X, Lu R, Zhang H, Ge J, Xiao B, et al. Hsa_circ_0065149 is an Indicator for Early Gastric Cancer Screening and Prognosis Prediction. Pathol Oncol Res. 2019.

24. Li WH, Song YC, Zhang H, Zhou ZJ, Xie X, Zeng QN, et al. Decreased Expression of Hsa_circ_00001649 in Gastric Cancer and Its Clinical Significance. Dis Markers. 2017;2017:4587698.

25. Lu R, Shao Y, Ye G, Xiao B, Guo J. Low expression of hsa_circ_0006633 in human gastric cancer and its clinical significances. Tumour Biol. 2017;39(6):1010428317704175.

26. Sun H, Tang W, Rong D, Jin H, Fu K, Zhang W, et al. Hsa_circ_0000520, a potential new circular RNA biomarker, is involved in gastric carcinoma. Cancer Biomark. 2018;21(2):299-306.

27. Tang W, Fu K, Sun H, Rong D, Wang H, Cao H. CircRNA microarray profiling identifies a novel circulating biomarker for detection of gastric cancer. Mol Cancer. 2018;17(1):137.

28. Sun H, Xi P, Sun Z, Wang Q, Zhu B, Zhou J, et al. Circ-SFMBT2 promotes the proliferation of gastric cancer cells through sponging miR-182-5p to enhance CREB1 expression. Cancer Manag Res. 2018;10:5725-34.

29. Huang M, He YR, Liang LC, Huang Q, Zhu ZQ. Circular RNA hsa_circ_0000745 may serve as a diagnostic marker for gastric cancer. World J Gastroenterol. 2017;23(34):6330-8.

30. Li T, Shao Y, Fu L, Xie Y, Zhu L, Sun W, et al. Plasma circular RNA profiling of patients with gastric cancer and their droplet digital RT-PCR detection. J Mol Med (Berl). 2018;96(1):85-96.

31. Chen LH, Wang LP, Ma XQ. Circ_SPECC1 enhances the inhibition of miR-526b on downstream KDM4A/YAP1 pathway to regulate the growth and invasion of gastric cancer cells. Biochem Biophys Res Commun. 2019;517(2):253-9.

32. Cai J, Chen Z, Zuo X. circSMARCA5 Functions as a Diagnostic and Prognostic Biomarker for Gastric Cancer. Dis Markers. 2019;2019:2473652.

33. Lu J, Zhang PY, Xie JW, Wang JB, Lin JX, Chen QY, et al. Hsa_circ_0000467 promotes cancer progression and serves as a diagnostic and prognostic biomarker for gastric cancer. J Clin Lab Anal. 2019;33(3):e22726.

34. Rong D, Lu C, Zhang B, Fu K, Zhao S, Tang W, et al. CircPSMC3 suppresses the proliferation and metastasis of gastric cancer by acting as a competitive endogenous RNA through sponging miR-296-5p. Mol Cancer. 2019;18(1):25.

35. Zhang H, Zhu L, Bai M, Liu Y, Zhan Y, Deng T, et al. Exosomal circRNA derived from gastric tumor promotes white adipose browning by targeting the miR-133/PRDM16 pathway. Int J Cancer. 2019;144(10):2501-15.

36. Zhao Q, Chen S, Li T, Xiao B, Zhang X. Clinical values of circular RNA 0000181 in the screening of gastric cancer. J Clin Lab Anal. 2018;32(4):e22333.

37. Han L, Zhang X, Wang A, Ji Y, Cao X, Qin Q, et al. A Dual-Circular RNA Signature as a Non-invasive Diagnostic Biomarker for Gastric Cancer. Front Oncol. 2020;10:184.

38. Lu J, Zhang PY, Xie JW, Wang JB, Lin JX, Chen QY, et al. Circular RNA hsa_circ_0006848 Related to Ribosomal Protein L6 Acts as a Novel Biomarker for Early Gastric Cancer. Dis Markers. 2019;2019:3863458.

39. Peng YK, Pu K, Su HX, Zhang J, Zheng Y, Ji R, et al. Circular RNA hsa_circ_0010882 promotes the progression of gastric cancer via regulation of the PI3K/Akt/mTOR signaling pathway. Eur Rev Med Pharmacol Sci. 2020;24(3):1142-51.

40. Lu J, Wang YH, Yoon C, Huang XY, Xu Y, Xie JW, et al. Circular RNA circ-RanGAP1 regulates VEGFA expression by targeting miR-877-3p to facilitate gastric cancer invasion and metastasis. Cancer Lett. 2020;471:38-48.

41. Tao X, Shao Y, Lu R, Ye Q, Xiao B, Ye G, et al. Clinical significance of hsa_circ_0000419 in gastric cancer screening and prognosis estimation. Pathol Res Pract. 2020;216(1):152763.

42. Li P, Chen S, Chen H, Mo X, Li T, Shao Y, et al. Using circular RNA as a novel type of biomarker in the screening of gastric cancer. Clin Chim Acta. 2015;444:132-6.

43. Guo W, Zhang J, Zhang D, Cao S, Li G, Zhang S, et al. Polymorphisms and expression pattern of circular RNA circ-ITCH contributes to the carcinogenesis of hepatocellular carcinoma. Oncotarget. 2017;8(29):48169-77.

44. Gong Y, Mao J, Wu D, Wang X, Li L, Zhu L, et al. Circ-ZEB1.33 promotes the proliferation of human HCC by sponging miR-200a-3p and upregulating CDK6. Cancer Cell Int. 2018;18:116.

45. Zhu K, Zhan H, Peng Y, Yang L, Gao Q, Jia H, et al. Plasma hsa_circ_0027089 is a diagnostic biomarker for hepatitis B virus-related hepatocellular carcinoma. Carcinogenesis. 2019.

46. Yu J, Ding WB, Wang MC, Guo XG, Xu J, Xu QG, et al. Plasma circular RNA panel to diagnose hepatitis B virus-related hepatocellular carcinoma: A large-scale, multicenter study. Int J Cancer. 2020;146(6):1754-63.

47. Li Z, Zhou Y, Yang G, He S, Qiu X, Zhang L, et al. Using circular RNA SMARCA5 as a potential novel biomarker for hepatocellular carcinoma. Clin Chim Acta. 2019;492:37-44.

48. Zhang H, Deng T, Ge S, Liu Y, Bai M, Zhu K, et al. Exosome circRNA secreted from adipocytes promotes the growth of hepatocellular carcinoma by targeting deubiquitination-related USP7. Oncogene. 2019;38(15):2844-59.

49. Lei B, Zhou J, Xuan X, Tian Z, Zhang M, Gao W, et al. Circular RNA expression profiles of peripheral blood mononuclear cells in hepatocellular carcinoma patients by sequence analysis. Cancer Med. 2019;8(4):1423-33.

50. Qiao GL, Chen L, Jiang WH, Yang C, Yang CM, Song LN, et al. Hsa_circ_0003998 may be used as a new biomarker for the diagnosis and prognosis of hepatocellular carcinoma. Onco Targets Ther. 2019;12:5849-60.

51. Wang G, Liu W, Zou Y, Wang G, Deng Y, Luo J, et al. Three isoforms of exosomal circPTGR1 promote hepatocellular carcinoma metastasis via the miR449a-MET pathway. EBioMedicine. 2019;40:432-45.

52. Sun S, Wang W, Luo X, Li Y, Liu B, Li X, et al. Circular RNA circ-ADD3 inhibits hepatocellular carcinoma metastasis through facilitating EZH2 degradation via CDK1-mediated ubiquitination. Am J Cancer Res. 2019;9(8):1695-707.

53. Chen W, Quan Y, Fan S, Wang H, Liang J, Huang L, et al. Exosome-transmitted circular RNA hsa_circ_0051443 suppresses hepatocellular carcinoma progression. Cancer Lett. 2020;475:119-28.

54. Weng Q, Chen M, Li M, Zheng YF, Shao G, Fan W, et al. Global microarray profiling identified hsa_circ_0064428 as a potential immune-associated prognosis biomarker for hepatocellular carcinoma. J Med Genet. 2019;56(1):32-8.

55. Zhou S, Wei J, Wang Y, Liu X. Cisplatin resistance-associated circRNA_101237 serves as a prognostic biomarker in hepatocellular carcinoma. Exp Ther Med. 2020;19(4):2733-40.

56. Zhang Y, Wang Q, Zhu D, Rong J, Shi W, Cao X. Up-regulation of circ-SMAD7 inhibits tumor proliferation and migration in esophageal squamous cell carcinoma. Biomed Pharmacother. 2019;111:596-601.

57. Wang Q, Zhang Q, Sun H, Tang W, Yang L, Xu Z, et al. Circ-TTC17 Promotes Proliferation and Migration of Esophageal Squamous Cell Carcinoma. Dig Dis Sci. 2019;64(3):751-8.

58. Fan L, Cao Q, Liu J, Zhang J, Li B. Circular RNA profiling and its potential for esophageal squamous cell cancer diagnosis and prognosis. Mol Cancer. 2019;18(1):16.

59. Hu X, Wu D, He X, Zhao H, He Z, Lin J, et al. circGSK3beta promotes metastasis in esophageal squamous cell carcinoma by augmenting beta-catenin signaling. Mol Cancer. 2019;18(1):160.

60. Huang E, Fu J, Yu Q, Xie P, Yang Z, Ji H, et al. CircRNA hsa_circ_0004771 promotes esophageal squamous cell cancer progression via miR-339-5p/CDC25A axis. Epigenomics. 2020.

61. Wang Q, Liu H, Liu Z, Yang L, Zhou J, Cao X, et al. Circ-SLC7A5, a potential prognostic circulating biomarker for detection of ESCC. Cancer Genet. 2020;240:33-9.

62. Tang X, Liu S, Ding Y, Guo C, Guo J, Hua K, et al. Serum Circular FoxO3a Serves as a Novel Prognostic Biomarker in Squamous Cervical Cancer. Cancer Manag Res. 2020;12:2531-40.

63. Yang C, Wei Y, Yu L, Xiao Y. Identification of Altered Circular RNA Expression in Serum Exosomes from Patients with Papillary Thyroid Carcinoma by High-Throughput Sequencing. Med Sci Monit. 2019;25:2785-91.

64. Wu G, Zhou W, Pan X, Sun Z, Sun Y, Xu H, et al. Circular RNA Profiling Reveals Exosomal circ_0006156 as a Novel Biomarker in Papillary Thyroid Cancer. Mol Ther Nucleic Acids. 2020;19:1134-44.

65. Xu ZQ, Yang MG, Liu HJ, Su CQ. Circular RNA hsa_circ_0003221 (circPTK2) promotes the proliferation and migration of bladder cancer cells. J Cell Biochem. 2018;119(4):3317-25.

66. Chi BJ, Zhao DM, Liu L, Yin XZ, Wang FF, Bi S, et al. Downregulation of hsa_circ_0000285 serves as a prognostic biomarker for bladder cancer and is involved in cisplatin resistance. Neoplasma. 2019;66(2):197-202.

67. Sun M, Zhao W, Chen Z, Li M, Li S, Wu B, et al. Circular RNA CEP128 promotes bladder cancer progression by regulating Mir-145-5p/Myd88 via MAPK signaling pathway. Int J Cancer. 2019;145(8):2170-81.

68. Chen X, Chen RX, Wei WS, Li YH, Feng ZH, Tan L, et al. PRMT5 Circular RNA Promotes Metastasis of Urothelial Carcinoma of the Bladder through Sponging miR-30c to Induce Epithelial-Mesenchymal Transition. Clin Cancer Res. 2018;24(24):6319-30.

69. Wang J, Wu A, Yang B, Zhu X, Teng Y, Ai Z. Profiling and bioinformatics analyses reveal differential circular RNA expression in ovarian cancer. Gene. 2020;724:144150.

70. Hu Y, Zhu Y, Zhang W, Lang J, Ning L. Utility Of Plasma circBNC2 As A Diagnostic Biomarker In Epithelial Ovarian Cancer. Onco Targets Ther. 2019;12:9715-23.

71. Wang W, Wang J, Zhang X, Liu G. Serum circSETDB1 is a promising biomarker for predicting response to platinum-taxane-combined chemotherapy and relapse in high-grade serous ovarian cancer. Onco Targets Ther. 2019;12:7451-7.

72. Zhao Z, Ji M, Wang Q, He N, Li Y. Circular RNA Cdr1as Upregulates SCAI to Suppress Cisplatin Resistance in Ovarian Cancer via miR-1270 Suppression. Mol Ther Nucleic Acids. 2019;18:24-33.

73. Li S, Pei Y, Wang W, Liu F, Zheng K, Zhang X. Extracellular nanovesicles-transmitted circular RNA has_circ_0000190 suppresses osteosarcoma progression. J Cell Mol Med. 2020;24(3):2202-14.

74. Xiao-Long M, Kun-Peng Z, Chun-Lin Z. Circular RNA circ_HIPK3 is down-regulated and suppresses cell proliferation, migration and invasion in osteosarcoma. J Cancer. 2018;9(10):1856-62.

75. Kun-Peng Z, Chun-Lin Z, Jian-Ping H, Lei Z. A novel circulating hsa_circ_0081001 act as a potential biomarker for diagnosis and prognosis of osteosarcoma. Int J Biol Sci. 2018;14(11):1513-20.

76. Kun-Peng Z, Xiao-Long M, Chun-Lin Z. Overexpressed circPVT1, a potential new circular RNA biomarker, contributes to doxorubicin and cisplatin resistance of osteosarcoma cells by regulating ABCB1. Int J Biol Sci. 2018;14(3):321-30.

77. Zhu K, Niu L, Wang J, Wang Y, Zhou J, Wang F, et al. Circular RNA hsa_circ_0000885 Levels are Increased in Tissue and Serum Samples from Patients with Osteosarcoma. Med Sci Monit. 2019;25:1499-505.

78. Yang F, Liu DY, Guo JT, Ge N, Zhu P, Liu X, et al. Circular RNA circ-LDLRAD3 as a biomarker in diagnosis of pancreatic cancer. World J Gastroenterol. 2017;23(47):8345-54.

79. Li J, Li Z, Jiang P, Peng M, Zhang X, Chen K, et al. Circular RNA IARS (circ-IARS) secreted by pancreatic cancer cells and located within exosomes regulates endothelial monolayer permeability to promote tumor metastasis. J Exp Clin Cancer Res. 2018;37(1):177.

80. Li Z, Yanfang W, Li J, Jiang P, Peng T, Chen K, et al. Tumor-released exosomal circular RNA PDE8A promotes invasive growth via the miR-338/MACC1/MET pathway in pancreatic cancer. Cancer Lett. 2018;432:237-50.

81. Shuai M, Hong J, Huang D, Zhang X, Tian Y. Upregulation of circRNA_0000285 serves as a prognostic biomarker for nasopharyngeal carcinoma and is involved in radiosensitivity. Oncol Lett. 2018;16(5):6495-501.

82. Guo Y, Huang Q, Zheng J, Hsueh CY, Huang J, Yuan X, et al. Diagnostic Significance of Downregulated circMORC3 as a Molecular Biomarker of Hypopharyngeal Squamous Cell Carcinoma: A Pilot Study. Cancer Manag Res. 2020;12:43-9.

83. Shuai M, Huang L. High Expression of hsa_circRNA_001387 in Nasopharyngeal Carcinoma and the Effect on Efficacy of Radiotherapy. Onco Targets Ther. 2020;13:3965-73.

84. Xu H, Gong Z, Shen Y, Fang Y, Zhong S. Circular RNA expression in extracellular vesicles isolated from serum of patients with endometrial cancer. Epigenomics. 2018;10(2):187-97.

85. Louis C, Desoteux M, Coulouarn C. Exosomal circRNAs: new players in the field of cholangiocarcinoma. Clin Sci (Lond). 2019;133(21):2239-44.

86. Jin C, Shi L, Li Z, Liu W, Zhao B, Qiu Y, et al. Circ_0039569 promotes renal cell carcinoma growth and metastasis by regulating miR-34a-5p/CCL22. Am J Transl Res. 2019;11(8):4935-45.
